# The impact of internship experience on professional identity, motivation, and attitude among aviation service majors: a cross-sectional empirical study

**DOI:** 10.3389/fpsyg.2025.1661068

**Published:** 2025-11-19

**Authors:** Fangming Wang

**Affiliations:** School of Art and Design, Jilin Engineering Normal University, Changchun, China

**Keywords:** aviation education, internship experience, professional identity, motivation, attitude, mediation, China

## Abstract

**Introduction:**

China’s rapidly expanding aviation industry has created an urgent need for a motivated and professionally committed workforce. This study examined whether internship experience influences aviation students’ career motivation and professional attitude through the mediating role of professional identity.

**Methods:**

A cross-sectional survey was conducted with 414 aviation service students using validated psychological measures. Mediation analysis was performed using PROCESS Model 4 with 5,000 bootstrap samples. Gender and first-choice major were included as covariates.

**Results:**

Higher-quality internship experiences significantly enhanced students’ professional identity, which strongly predicted both career motivation (*β* = 0.50***) and professional attitude (*β* = 0.48***). Professional identity partially mediated the effect of internship experience on career motivation (indirect effect = 0.30) and fully mediated its effect on professional attitude (indirect effect = 0.24**).

**Discussion:**

These findings demonstrate that internships shape students’ long-term career outcomes primarily by strengthening their professional identity rather than through direct experiential effects alone. Professional identity is therefore a critical psychological mechanism in aviation talent development, emphasizing the need for identity-supportive internship programs.

## 1 Introduction

Aviation service management education in China has rapidly expanded, responding to the growing demand for skilled aviation professionals amid significant growth in the aviation industry (Yiu et al., 2022). However, despite increased enrollment and curriculum development, the gap between academic preparation and actual workplace expectations remains substantial (O'Brien and Bates, 2015). The professional identity, motivation, and attitudes of aviation service students—critical factors influencing career commitment and long-term professional success—have not been adequately studied, particularly concerning practical experiences such as internships (Watts et al., 2024).

Professional identity refers to how individuals define themselves within the context of their occupation, profoundly shaping their career decisions and persistence in the profession (Toubassi et al., 2023). Previous studies have highlighted that well-developed professional identity significantly correlates with higher job satisfaction, increased career motivation, and reduced turnover intention (Zhang et al., 2018; Zheng et al., 2025). In aviation service management, where rigorous standards, service orientation, and safety awareness are paramount, cultivating a robust professional identity during education is essential. Career motivation, driven by both intrinsic factors (e.g., passion for aviation service, personal satisfaction) and extrinsic factors (e.g., career stability, salary), also plays a crucial role in determining student engagement and career outcomes (Schlichting, 2022). Students who possess strong career motivation are more likely to pursue continuous professional growth and demonstrate resilience in challenging work environments. However, motivation alone may not suffice; attitudes toward the profession—reflecting students’ perceptions, concerns, and expectations—significantly influence their career trajectories (Isnaini et al., 2021). Positive attitudes help students adapt effectively to workplace challenges, whereas negative or uncertain attitudes may lead to dissatisfaction and career withdrawal (Ventista and Brown, 2023).

Internships, providing practical exposure to industry environments, are integral to vocational education. High-quality internship experiences enable students to connect theoretical knowledge with practical skills, foster professional identity development, and strengthen career motivation (Gerçek and Özveren, 2024). Yet, the direct impact of internship experiences on aviation service students’ professional identity, motivation, and attitudes remains unclear, particularly within the context of China’s rapidly evolving aviation sector (Wan et al., 2011; Pach et al., 2025; Sholikhah et al., 2025).

Therefore, this study aims to explore the relationships among internship experiences, professional identity, career motivation, and professional attitudes among aviation service management students in China. Specifically, it investigates how internship experiences influence professional identity formation, how identity subsequently shapes career motivation and attitudes, and ultimately, how these factors collectively impact students’ career readiness. The findings of this research will provide educators and policymakers with valuable insights for designing curricula and internship programs that better align educational experiences with industry expectations, thereby enhancing students’ career outcomes and meeting the aviation industry’s workforce demands.

While there is extensive literature on internships and identity development in fields like teacher training and nursing, the aviation education context remains under-explored. Aviation service majors (typically students in programs for airline hospitality, cabin crew training, or airport operations) represent a unique population of vocational students. They often undergo intensive practical training and mandatory internships with airlines or airports as part of their curriculum (Chen et al., 2020). The extent to which these internships contribute to forming an “airline professional” identity, and how that impacts their motivation and attitude, is a question of both theoretical and practical significance. By investigating these links, our study seeks to extend the general theory of professional identity development to a new context and provide guidance to aviation education stakeholders on designing effective training.

Research Purpose and Questions: In summary, this study aims to examine the impact of internship experience on professional identity among aviation service majors, and how professional identity subsequently influences students’ motivation and attitude toward their career. We posit a mediation framework in which internship experiences (independent variable) strengthen students’ professional identity (mediator), leading to higher motivation and more positive professional attitudes (dependent outcomes).

The research is guided by the following questions:

1. Does participation in aviation service internships significantly enhance students’ professional identity?
2. Is professional identity associated with greater motivation and more positive career attitudes among these students?
3. Does professional identity mediate the relationship between internship experience and students’ motivation/attitude?

By answering these questions, we hope to illuminate the psychological mechanisms through which real-world training experiences shape aviation students’ professional development.

## 2 Literature review

### 2.1 Professional identity and student motivation in vocational education

Professional identity broadly refers to an individual’s perception, values, and beliefs about belonging to a professional community. It is shaped significantly through educational experiences and practical engagement within a chosen field (Olsen et al., 2024). Prior research consistently indicates that a robust professional identity positively influences student motivation and engagement across various vocational fields, notably nursing and teacher education (Gao et al., 2025; Zou et al., 2024). For instance, students with clearer professional identities tend to demonstrate higher motivation, initiative, and sustained engagement in their studies (Namaziandost et al., 2024).

Career motivation in vocational education encompasses intrinsic factors, such as personal satisfaction and passion for the profession, and extrinsic factors, including career stability and financial rewards (Chin et al., 2020). Empirical evidence from nursing education suggests that professional identity significantly predicts higher motivation and innovative capabilities, with motivation often serving as a mediating variable that enhances performance outcomes (Faihs et al., 2023). Similarly, studies in teacher education have demonstrated that a strong professional identity correlates with increased resilience, reduced burnout, and higher retention rates within the profession (Pi et al., 2024).

Internship experiences play a crucial role in the development of professional identity, particularly in vocational training contexts (O'Brien and Bates, 2015). Internships enable students to bridge theoretical knowledge with practical experience, often solidifying their identification with their intended profession (Oyserman, 2015). For instance, nursing and teaching practicums significantly enhance students’ professional identities by providing authentic, immersive work experiences and mentor support (Oyserman et al., 2014a). Conversely, poor-quality internships can negatively impact professional identity, potentially deterring students from continuing in their respective fields (Tagawa, 2020).

Within aviation service education, internships typically involve direct exposure to operational roles, such as passenger interactions, adherence to safety and service standards, and managing challenging situations. These practical experiences are hypothesized to be instrumental in forming a clear and stable professional identity among students (Xu et al., 2023).

This study specifically investigates the mediating role of professional identity between internship experiences and key educational outcomes, including motivation and professional attitudes. Drawing on theories such as Social Cognitive Career Theory (SCCT) (Ahmed et al., 2024), we propose that internship experiences positively affect students’ professional identities, which in turn enhance their motivation and foster more positive attitudes toward their careers. Thus, this research aims to provide empirical insights and practical guidance for structuring internships within aviation education programs to optimize professional identity formation and subsequent motivational outcomes.

### 2.2 Internship experiences and professional identity formation

Internship or practicum experiences are widely regarded as crucial for professional identity development, especially in pre-professional contexts where students transition from classroom learning to practical application (Cai et al., 2022). In nursing education, the clinical internship is identified as a “key period for nursing students to form professional identity” (Mann et al., 2009). During this period—often the final year of training—students work under supervision in hospitals or clinics, which enables them to reconcile theoretical knowledge with real patient-care experiences. Research indicates that through internships, nursing students compare their “imagined practice with reality,” become familiar with actual work environments, and thus develop a more realistic and internalized professional identity (Muthanna and Khine, 2023).

Some studies further suggest that only by performing job tasks and navigating authentic workplace challenges can students determine whether they truly “fit” the profession, thereby solidifying or reshaping their identity (Sylvia et al., 2023). Pianda et al. (2024) identified several key factors during internships that influence the development of professional identity, including the organizational atmosphere and quality of mentorship (Pianda et al., 2024). A supportive environment and effective clinical instructors were associated with higher professional identity scores among interns, highlighting that the quality of the internship experience—not just its existence—plays a pivotal role in shaping identity.

Likewise, in teacher education, the teaching practicum is often viewed as a transformative experience in shaping professional identity (Field, 2011). Numerous studies have documented significant changes in preservice teachers’ self-perception and role internalization following internship experiences (Niittylahti et al., 2021). Beyond education and health, similar patterns have been observed in other pre-professional contexts (Ford et al., 2024). In engineering education, internships can either reinforce or disrupt students’ emerging professional identities. For some students—particularly those from underrepresented backgrounds—negative experiences such as exclusion or stereotype bias may lead them to question their fit in the field. Trede et al. (2012), for instance, found that women of color in engineering programs reported stronger professional identity development when their internships provided inclusive environments and meaningful project engagement (Trede et al., 2012). Conversely, when faced with microaggressions or limited involvement, their sense of belonging and professional self-concept was weakened.

In hospitality and tourism management, internships at hotels or airlines often act as a “reality check” for students’ expectations versus workplace realities (Phillips, 2016). When there is alignment between anticipated and actual work experiences, students tend to develop stronger commitment and identity with the profession. However, if the internship is of poor quality or lacks sufficient challenge, it may discourage continued interest in the career path. Thus, internship outcomes for identity formation are shaped not only by the structural elements of the experience but also by the student’s personal interpretation and reflection within the host organization’s context (Jackson, 2016).

### 2.3 Professional identity as a mediator of internship effects on motivation and attitude

The theoretical framework guiding this study proposes that professional identity serves as a mediating variable linking internship experiences to key student outcomes—namely, career motivation and professional attitude (Cai et al., 2022). In other words, we hypothesize that internships affect students’ motivation and attitudes indirectly, by first shaping their professional identity. This mediation hypothesis is supported by prior studies that have examined sequential relationships among similar constructs. For instance, Wang et al. (2025) found that professional identity mediated the relationship between achievement motivation and research ability among nursing interns (Wang et al., 2025). Students with higher motivation tended to develop stronger research capabilities, and part of this effect was explained by increased identification with their profession—suggesting that professional identity helps transform motivation into actual performance.

By parallel reasoning, one could argue that an effective internship may enhance a student’s identification with the aviation profession, which in turn elevates their motivation and fosters more positive professional attitudes. Rather than acting directly on motivation or attitude, the internship exerts its influence through identity development.

From a social-cognitive perspective, this mediation pathway aligns with Social Cognitive Career Theory (SCCT), which posits that real-world accomplishments (e.g., successful internships) build self-efficacy and shape outcome expectations, which then influence career goals and persistence (Liao et al., 2024). Professional identity can be considered part of a learner’s cognitive-affective profile—encompassing self-efficacy, a sense of belonging, and internalized values. A well-designed internship may strengthen a student’s belief in their competence (“I can do this job”) and simultaneously deepen their value alignment (“I am a flight attendant at heart”), thereby enhancing both motivation and resilience.

Educational psychology also reinforces this view: attitudes play a central role in guiding behavior, and professional identity—as a specific attitudinal stance toward one’s career—can profoundly impact what students choose to learn and how they engage with tasks (Lewis et al., 2023). Empirical findings in healthcare education further support this view. A 2025 study by Y. Yuan revealed that professional identity and a sense of coherence jointly mediated the relationship between compassion fatigue and work engagement among hospital nurses in China (Yuan et al., 2025). Applied to aviation students, this suggests that a challenging internship—while potentially stressful—can promote positive outcomes if it strengthens identity (“I am a capable aviation professional”). However, if identity fails to develop, the same stressors may lead to demotivation and negative attitudes.

**Figure 1:**
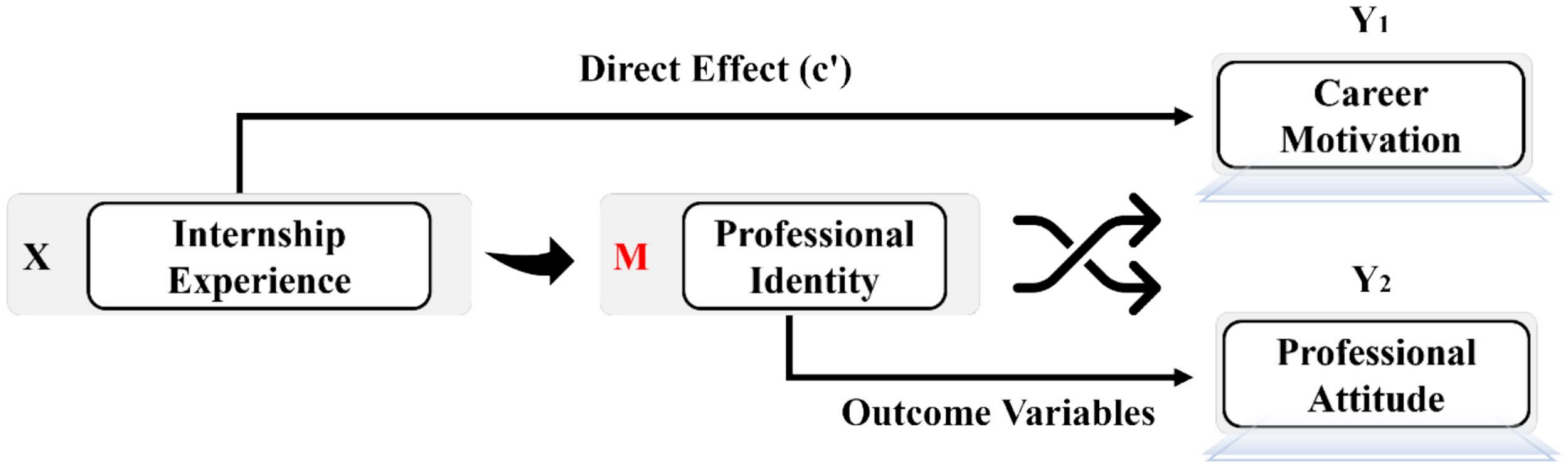
Conceptual framework of the mediating role of professional identity.

Collectively, the reviewed literature suggests that internship experiences play a critical role in shaping aviation students’ professional development. However, prior studies have primarily focused on direct outcomes of internships and have paid limited attention to the psychological processes through which these outcomes occur. Drawing on identity theory and empirical evidence from vocational education, this study advances the proposition that professional identity functions as a psychological bridge that channels internship experience into long-term motivational and attitudinal outcomes. Accordingly, we propose a conceptual framework in which internship experience influences career motivation and professional attitude indirectly through the formation of professional identity. The hypothesized model is presented in Figure 1. Our conceptual model—grounded in mediation theory and supported by prior literature—posits the following sequence: Internship Experience → Professional Identity → Motivation and Attitude. We anticipate that students who have completed internships will report higher levels of professional identity compared to those without such experiences, and that identity will significantly mediate the relationship between internship participation and both motivation and career attitude. This framework offers new insight into how identity acts as a bridge between practical training and educational outcomes, and it has practical implications for optimizing aviation training programs.

## 3 Methodology

### 3.1 Research design

We employed a cross-sectional survey design for this empirical study. Data were collected at a single time point (from July 4 to September 20, 2024, during the summer semester break) through structured questionnaires administered to aviation service management students. The survey aimed to capture students’ internship experiences, professional identity, motivation, and career attitudes. This study is correlational and explanatory in nature, focusing on the hypothesized mediating role of professional identity between internship experience and student outcomes. Given the theoretical orientation and self-reported nature of the constructs, a cross-sectional design was deemed appropriate for measuring perceptions after students’ internship completion. The design and reporting of this study followed established best practices in survey-based educational research.

### 3.2 Participants

The target population consisted of students enrolled in aviation service education programs, including majors such as Airline Cabin Crew Training, Aviation Hospitality, and Airport Ground Services. The study focused on students in the later stages of their programs who had completed, or were in the process of completing, an internship as part of their academic curriculum. A convenience sample was drawn from three institutions in China: Jilin Normal University of Engineering and Technology, Changchun University of Technology, and Changchun Normal University College of Humanities. Program administrators and teaching faculty from these institutions assisted in distributing the survey to eligible students via class communication channels.

In total, 450 questionnaires were distributed, and 414 valid responses were received and included in the final analysis (response rate ≈ 92%). Among the respondents, approximately 70% were female and 30% male, a distribution that reflects the gender composition typically found in cabin crew and hospitality training cohorts. Participants ranged in age from 19 to 24 years (*M* ≈ 22). About 90% of students were in their final year and had recently completed a mandatory internship at an airline or airport (typically lasting 3–6 months), while approximately 10% were third-year students who had just begun or were about to begin their internship.

To ensure meaningful responses, only students with internship experience (either completed or in progress) were included in analyses related to internship effects. This sampling strategy captured a relevant cross-section of pre-professional aviation service students currently transitioning from education to full-time employment in the aviation industry.

### 3.3 Instrumentation

Data were collected using a structured, self-administered questionnaire comprising several standardized scales. The instrument was adapted from an existing survey originally developed for English teacher trainees and revised to reflect the aviation service context. Specifically, references to teaching were replaced with aviation-relevant terms (e.g., “teaching internship” became “aviation service internship,” “students/pupils” became “passengers/customers,” and “teaching career” was replaced by “airline career”). The adaptation process was reviewed by two experts—one in teacher education and one in aviation service training—to ensure both the psychometric integrity of the scales and the contextual relevance of the wording for aviation students.

The final questionnaire consisted of the following sections:

Internship Experience Scale. This section assessed the perceived quality and characteristics of students’ internship experiences. It was adapted from validated instruments used in teacher education research, such as Sun and Van Es (2015), and included items assessing supervision (e.g., “My internship mentor or supervisor was supportive and provided regular feedback”), work environment (e.g., “The airline or airport work atmosphere was welcoming for trainees”), opportunities to apply knowledge, and overall satisfaction. Students responded on a 5-point Likert scale ranging from 1 (strongly disagree) to 5 (strongly agree). A composite score was calculated by averaging all items, with higher scores indicating more enriching internship experiences. This variable served as the independent variable in the mediation model and was also used for group comparisons.

Professional Identity Scale. Professional identity was measured using an adapted version of Brown et al. (1986) Professional Identification Scale, which has previously been validated in nursing education contexts (Brown et al., 1986). The scale encompasses cognitive, affective, and behavioral dimensions of professional identity. Example items include: “I am proud to be in the aviation service profession,” “I see myself as an aviation service professional,” and “Being a cabin crew or ground staff member is an important part of who I am.” Responses were recorded on a 5-point Likert scale. Higher scores indicated stronger identification with the aviation profession. Internal consistency in the current sample was high (Cronbach’s *α* = 0.85), suggesting good reliability. This construct served as the mediator in our conceptual model.

Motivation Scale. Students’ motivation toward aviation-related study and work was measured using items adapted from the Revised Life Goals Questionnaire and intrinsic motivation scales tailored to vocational careers (Ryan and Deci, 2000). Items included: “I am highly motivated to acquire the skills needed for an airline service job,” “I feel driven to excel in this field,” and “I would work hard in this career even if the job is challenging.” Responses were captured using a 5-point Likert scale. While a few behavioral engagement items (e.g., time spent on extra practice, number of relevant certifications obtained) were included for descriptive purposes, the composite motivation score (average of main items) was used as a dependent variable in mediation testing.

Professional Attitude Scale. To measure students’ attitudes toward the aviation service profession, we adapted items from nursing career commitment surveys (Zhao et al., 2022). This short scale covered dimensions such as long-term commitment (e.g., “I am committed to a long-term career in aviation service”), value congruence (e.g., “I believe in the values of the aviation service industry, such as safety and teamwork”), and turnover intention (reverse-coded; e.g., “I have seriously considered leaving the aviation field for another career”). Higher composite scores reflected more positive and committed professional attitudes.

In addition to the main constructs, the questionnaire collected demographic and background information including: age, gender, program type and year of study, internship status (completed or ongoing), internship duration, and internship host organization type (e.g., domestic airline, international airline, airport ground services). Participants were also asked whether aviation had been their first-choice major, as previous studies suggest that first-choice status can influence professional identity levels (Zeng et al., 2022).

The original questionnaire was developed in Chinese and then translated into English using a back-translation procedure to ensure semantic accuracy. A pilot test involving 10 aviation students was conducted to evaluate clarity, relevance, and time to completion. Minor revisions were made based on feedback (e.g., clarifying that “aviation service profession” includes both cabin crew and ground operations). The average time to complete the questionnaire was approximately 15 min.

### 3.4 Data collection procedure

Data were collected between July 4 and September 20, 2024, during the final phase of the semester break, when most students had completed or nearly completed their internship placements. This timing ensured that participants’ responses reflected their full internship experiences rather than preliminary impressions.

Prior to data collection, ethical approval was obtained from the university’s academic review committee, and permissions were secured from program directors of the participating institutions. The survey was administered in both online and paper-based formats across three universities in Northeast China.

At Jilin Engineering Normal University, participants completed the questionnaire in classroom sessions using printed forms, which were collected immediately on site. At Changchun University of Technology and Changchun Normal University (College of Humanities), students received a secure online link through a widely used academic survey platform to complete the same questionnaire electronically.

Participation in the study was entirely voluntary, and anonymity was strictly maintained. No names, student identification numbers, or personally identifying information were collected. Prior to participation, all students provided informed consent, acknowledging that their responses would be used exclusively for research purposes and would not influence their academic evaluations.

For the paper-based surveys, trained research assistants were present in the classroom to answer clarifying questions and to ensure that students could complete the questionnaire privately. Immediately after collection, completed surveys were screened for missing responses; students who had unintentionally skipped items were asked to complete them in accordance with IRB-approved data collection protocols (Dillman et al., 2014). The online version of the survey, delivered via Wenjuanxing, included automatic checks for item completion and allowed participants to withdraw at any time.

In total, approximately 450 students were invited to participate, of whom 426 submitted responses. After data cleaning—removing incomplete submissions and responses that failed embedded attention-check items—a total of *N* = 414 valid responses remained for analysis, yielding an effective response rate of approximately 92%.

### 3.5 Data analysis plan

All statistical analyses were conducted using IBM SPSS Statistics (Version 26.0) along with Hayes’ PROCESS macro (Model 4) for mediation analysis. We began with descriptive statistics for all variables, including means, standard deviations, and normality checks. Internal consistency of each scale was assessed using Cronbach’s alpha to evaluate reliability.

To explore relationships among the key constructs—internship experience, professional identity, career motivation, and professional attitude—we first calculated Pearson correlation coefficients. These foundational associations informed subsequent analyses.

To address our core research questions, we implemented the following analytic procedures:

#### 3.5.1 Internship → professional identity

We conducted independent-samples *t*-tests and one-way ANOVAs to compare professional identity scores across students with different internship statuses (e.g., completed vs. in progress). Additionally, Pearson correlations were used to examine the association between internship quality (treated as a continuous variable) and professional identity. We hypothesized a significant positive correlation, suggesting that more enriching internship experiences predict stronger professional identity.

#### 3.5.2 Professional identity → motivation/attitude

To test whether professional identity predicts career motivation and professional attitude, we used Pearson correlations and simple linear regression. We also performed multiple regression analyses controlling for internship experience and demographic variables (e.g., gender, first-choice major status) to assess the unique contribution of identity.

#### 3.5.3 Mediation analysis

The primary model tested was a mediation structure in which internship experience (X) affects both motivation (Y₁) and professional attitude (Y₂) through professional identity (M). We used PROCESS macro (Model 4) with 5,000 bootstrap samples to estimate indirect effects and obtain bias-corrected 95% confidence intervals. Gender and whether aviation was the student’s first-choice major were included as covariates, based on prior findings showing their influence on identity and motivation. A significant indirect effect, along with a reduced direct effect when identity is included in the model, would support our mediation hypothesis.

We conducted subgroup analyses by internship quality (using a median split to compare high vs. low quality experiences) to explore whether the relationship between internship and identity differs in magnitude across groups. Additionally, we collected qualitative responses to an open-ended question inviting students to describe the most impactful aspect of their internship; selected themes will be used to contextualize the quantitative findings.

To assess potential common method bias, Harman’s single-factor test was conducted. The unrotated factor solution yielded four factors, with the first factor accounting for 27.4% of the total variance, well below the critical threshold of 40% (Podsakoff et al., 2003). Additionally, all VIF values were below 3.3, indicating that multicollinearity and common method variance are not a significant concern. All inferential analyses were conducted at a significance level of *α* = 0.05. Final results will be presented with corresponding tables, including descriptive statistics, correlation matrices, regression summaries, and mediation coefficients.

## 4 Results

This study aimed to examine the relationships among internship experience, professional identity, career motivation, and professional attitude in aviation service students. Descriptive statistics and reliability analyses were conducted to assess the overall trends and the psychometric quality of the measures.

As summarized in Table 1, students reported relatively high levels of professional identity, career motivation, and professional attitude, with all means exceeding the midpoint of the 5-point scale. The average professional identity score was 4.10 (SD = 0.57), suggesting a strong sense of identification with the aviation profession among respondents. Similarly, motivation (*M* = 4.02, SD = 0.60) and attitude (*M* = 3.95, SD = 0.68) scores reflected generally positive career orientations. Standard deviations across variables were moderate, indicating sufficient variability for further analysis. Notably, motivation scores showed slightly greater dispersion, suggesting some individual differences in students’ career drive and goal pursuit.

**Table 1:** Descriptive statistics for key study variables (*N* = 414).

Variable	Mean	SD	Scale range
Professional identity (Mediator) – 6 items	4.10	0.57	1–5
Career motivation (DV1) – 10 items	4.02	0.60	1–5
Professional attitude (DV2) – 5 items	3.95	0.68	1–5

Internship experience quality was only assessed for students who had completed an internship (*N* = 242). Higher scores indicate a more positive, enriching internship.

Internship experience quality was assessed only for the subset of participants who had completed an internship (*n* = 242). On average, these students reported a positive experience, with most items scoring above 3.5 on the 5-point scale (see further analysis in Table 2). These findings align with prior research suggesting that real-world exposure in aviation settings strengthens students’ perceptions of professional development opportunities.

**Table 2:** Exploratory factor analysis results for key constructs.

Item	Factor 1 identity	Factor 2 motivation	Factor 3 attitude
*“I am proud to be in the aviation service profession.”*	0.78	0.10	0.05
*“Being an airline service professional is important to who I am.”*	0.81	0.08	0.06
*“I see myself as an aviation service professional.”*	0.75	0.14	0.07
*“I am highly motivated to acquire skills for an airline job.”*	0.09	0.77	0.10
*“I feel driven to excel in this field.”*	0.11	0.80	0.12
*“I would work hard even if the job is challenging.”*	0.06	0.74	0.15
*“I am committed to a long-term career in aviation service.”*	0.05	0.13	0.82
*“I believe in the values of the aviation industry.”*	0.07	0.18	0.78
*“I have seriously considered leaving this field.”* (R)	0.02	0.10	0.75

Loadings < 0.20 are suppressed for clarity. Each item is abbreviated; (R) denotes a reverse-coded item. The three extracted factors accounted for a cumulative 67% of the variance, with Factor 1 (Identity) explaining 28%, Factor 2 (Motivation) 22%, and Factor 3 (Attitude) 17%. The high KMO value and significant Bartlett’s test indicate that the data were suitable for factor analysis, and the rotated solution aligns with the theorized dimensions of Identity, Motivation, and Attitude.

To evaluate the internal consistency of the scales, Cronbach’s alpha coefficients were computed. As shown in Table 3, all multi-item scales demonstrated acceptable to excellent reliability. Cronbach’s alpha values ranged from 0.78 to 0.88, exceeding the widely accepted threshold of 0.70. These results indicate that the items for each construct reliably capture the intended latent variables among aviation service students.

**Table 3:** Scale reliability analysis (Cronbach’s α).

Scale	Number of items	Cronbach’s α
Professional identity	6	0.85
Career motivation	10	0.88
Professional attitude	5	0.78

### 4.1 Internship experiences enhance professional identity

Consistent with prior research in education and health fields, the current study confirms that aviation internships play a significant role in shaping students’ professional identity. Students who had completed an internship reported significantly higher professional identity scores compared to those who had not, and the perceived quality of internship experience was positively correlated with identity strength (see correlation matrix in Table 4). These findings align with studies in nursing education that identify the clinical internship as a critical phase for identity development (Zeng et al., 2022). In our aviation context, students likely encountered real-world tasks such as interacting with passengers, following safety protocols, and navigating team-based service challenges—experiences that help them internalize the role of an aviation professional.

**Table 4:** Pearson correlations among internship experience, professional identity, motivation, and attitude.

Variable	1. Internship experience	2. Prof. identity	3. Career motivation	4. Professional attitude
1. Internship experience	—			
2. Professional identity	0.32***	—		
3. Career motivation	0.25***	0.56***	—	
4. Professional attitude	0.20**	0.50***	0.45***	—

*N* = 414 (for Identity, Motivation, Attitude). Internship Experience quality was assessed only for the subset of students who completed an internship (n = 242); correlations involving Internship Experience use that subsample. All correlations are significant: *p* < 0.05; *p < 0.01; **p < 0.001 (two-tailed).

Qualitative comments from students (collected via an open-ended survey item) frequently highlighted key moments that made them “feel like cabin crew,” such as managing a delayed boarding or resolving a passenger complaint. These transformative moments mirror teacher education studies, where preservice teachers describe a shift in identity after leading a classroom or handling a student conflict (Cai et al., 2022).

To validate the structure of our survey instrument, an exploratory factor analysis (EFA) was conducted using principal component analysis with varimax rotation. The Kaiser–Meyer–Olkin (KMO) measure was 0.90, and Bartlett’s test of sphericity was significant [χ^2^(210) = 1945.3, *p* < 0.001], indicating suitability for factor analysis. As shown in Table 2, three distinct factors were extracted, corresponding to the theorized constructs: Professional Identity, Career Motivation, and Professional Attitude. All items loaded strongly on their intended factor (≥ 0.70), with minimal cross-loadings (all < 0.20, suppressed for clarity). This clear three-factor structure supports the construct validity of our adapted scales.

These findings support the hypothesis that internships bridge the gap between theory and practice, facilitating the transformation from student to professional. According to social identity theory, immersion in uniformed roles, adherence to codes of conduct, and being treated as “one of the crew” during internships can activate internalization of professional norms (Saks et al., 2007).

Crucially, the positive correlation between internship quality and professional identity underscores that the structure and supportiveness of the internship matter more than its mere completion. Students who rated their mentors and environments highly were more likely to report confident professional identities. This echoes findings in teacher education that mentor affirmation promotes the formation of a robust self-concept (Izadinia, 2016). Conversely, students in low-quality internships may develop only a fragile sense of professional identity—or even disengage. Fortunately, in our sample, very few participants reported negative experiences, possibly due to strong university–industry partnerships.

Taken together, these results reinforce conclusions from other domains—nursing, teaching, and hospitality—that internships are a formative crucible for professional identity development (McDonald and Wilson-Mah, 2022). For aviation education providers, the implication is clear: partnerships with airlines and airports must prioritize not only placement quantity but also mentorship quality and role immersion. When interns feel included and impactful, their identification with the profession grows stronger, laying a foundation for future motivation and career resilience.

### 4.2 Professional identity drives motivation and positive attitudes

A central contribution of this study is empirical evidence that professional identity is strongly linked to students’ motivation and career attitudes in the aviation service domain. As shown in Table 4, Pearson correlation coefficients reveal that professional identity was positively and significantly associated with both career motivation (*r* = 0.56, *p* < 0.001) and professional attitude (*r* = 0.50, *p* < 0.001). These findings suggest that students who more strongly identify with the aviation profession tend to exhibit greater internal drive and stronger commitment to remaining in the field.

This result aligns with prior research in nursing and teacher education, where stronger professional identity has been associated with “higher motivation, reduced learning fatigue, and sustained engagement” in both academic and clinical training (Zhang et al., 2023). In our sample, students who agreed with statements like “Being an airline service professional is important to who I am” were also more likely to express sentiments such as “I am highly motivated to acquire skills for this field” and “I am committed to a long-term career in aviation.”

From a theoretical standpoint, these results support identity-based motivation theory, which posits that when individuals internalize a professional identity, they become more motivated to act in ways congruent with that identity (Oyserman et al., 2014b). For example, an aviation student who views themselves as a future flight attendant may voluntarily engage in professional development activities—such as extra simulator training or language certification—because these actions affirm their role-based self-concept.

Multiple regression analyses further clarified the unique contribution of professional identity when controlling for internship experience. As shown in Table 5, professional identity was a strong and significant predictor of both motivation (*β* = 0.50, *p* < 0.001) and attitude (*β* = 0.48, *p* < 0.001), while the direct effect of internship experience was smaller or non-significant. Specifically, internship experience predicted motivation modestly (*β* = 0.15, *p* < 0.05) but had no significant direct effect on attitude when identity was included in the model (*β* = 0.05, ns). These models explained substantial variance: *R*^2^ = 0.34 for motivation and *R*^2^ = 0.26 for attitude, underscoring that professional identity is the primary psychological mechanism driving student engagement and outlook.

**Table 5:** Multiple regression analyses predicting motivation and attitude from internship experience and professional identity.

Predictor	**Motivation (DV1)**		**Attitude (DV2)**	
	***B* (SE)**	***β***	***B* (SE)**	***β***
Internship experience	0.21(0.09)*	0.15*	0.07 (0.08)	0.05 (n.s.)
Professional identity	0.59(0.09)***	0.50 ***	0.51.(0.08)***	0.48***
Constant	1.18(0.31)***	—	1.28 (0.29)***	—
*R*^2^	0.34		0.26	
Adjusted *R*^2^	0.33		0.25	
F (df₁, df₂)	76.5*** (2, 297)		52.3*** (2, 297)	

Unstandardized coefficients (B) with standard errors in parentheses; β = standardized coefficient. *p* < 0.05; *p < 0.01; **p < 0.001. The regression models control.

These results suggest that the positive effects of internship experiences on motivation and attitude operate primarily through identity enhancement—a classic mediation pattern. Similar dynamics have been observed in other domains: for example, in nursing, students with low identity scores are more likely to consider leaving the profession, while those with stronger identity report higher retention intentions. In our aviation cohort, students with high identity consistently expressed intent to stay in the field, while those with weaker identity were more likely to consider alternative careers.

This is particularly relevant in aviation, where cabin crew and service roles can appear glamorous but are often associated with irregular hours and emotional labor. A strong professional identity may buffer against early attrition, as it helps students internalize the realities of the job while maintaining a positive and resilient career mindset (Pires, 2023).

Our findings align with education psychology research suggesting that identity mediates the link between experience and motivation. For instance, Liu et al. (2023) found that professional identity predicted learning engagement among Chinese engineering students (Liu et al., 2023). These cross-domain parallels reinforce the notion that professional identity is a universal construct that shapes how students respond to training, challenges, and career preparation.

### 4.3 Mediation: professional identity as the link between internship and outcomes

One of the central theoretical objectives of this study was to test whether professional identity mediates the relationship between internship experience and two student outcomes: career motivation and professional attitude. As shown in Table 6, our PROCESS Model 4 analysis supports this hypothesis. Specifically, the effect of internship experience on both outcomes was significantly transmitted through professional identity.

**Table 6:** Mediation analysis summary: identity as a mediator between internship experience and outcomes.

Outcome (Y)	Total effect of internship	Direct effect controlling identity	Indirect effect (a × b via identity)	95% Bootstrap CI for indirect
Career motivation	0.45** (*p* = 0.004)	0.15* (p = 0.021)	0.30**	[0.18, 0.45]
Professional attitude	0.29* (*p* = 0.034)	0.05 (n.s., p = 0.35)	0.24**	[0.12, 0.40]

Unstandardized coefficients reported. Bootstrap CIs based on 5,000 resamples. Indirect effect is significant when CI does not include zero. *p* < 0.05 (), *p* < 0.01 (**).

For career motivation, the indirect effect (a × b) was 0.30, with a 95% bias-corrected bootstrap confidence interval of [0.18, 0.45], which does not include zero—indicating a significant mediating effect. The direct effect (c′) remained significant but smaller (*B* = 0.15, *p* = 0.021), consistent with partial mediation. For professional attitude, the indirect effect via identity was 0.24, with CI [0.12, 0.40], and the direct effect of internship experience dropped to non-significance (*B* = 0.05, *p* = 0.35), indicating full mediation.

These results suggest that the influence of internship experience on motivation and attitude operates primarily through its impact on students’ professional identity. This supports the theorized developmental chain: real-world experience → identity formation → psychological outcome.

To illustrate, consider two students: one who completed a structured, supportive internship with meaningful tasks and feedback (Student A), and one who completed a minimal, passive internship (Student B). Student A likely developed a strong identity (“I am meant for this job”) and thus showed higher motivation and stronger commitment to an aviation career. In contrast, Student B may have lacked such internalization and, consequently, reported lower engagement. Our statistical results reflect this narrative: internship quality correlated with outcomes, but identity explained much of the relationship.

These findings are consistent with prior mediation research. For example, Jiayi Zhu et al. (2024) demonstrated that professional identity mediated the link between motivation and research ability among nursing students (Zhu et al., 2024). While their model emphasized how internal motivation translates into performance via identity, our model demonstrates how external experience (internship) drives internal motivation via identity, essentially reversing the directional flow.

Theoretically, this supports calls to include professional identity as a core variable in models of vocational learning and development. Traditional motivational theories often focus on self-efficacy, values, and goals. Our results indicate that identity is a distinct construct that absorbs and transmits the impact of experiential learning. It also echoes the idea of “identity capital”: that students accumulate psychological resources—such as confidence, clarity, and role congruence—through identity development, which in turn fuels motivation and resilience.

## 5 Discussion

To situate the present findings within the broader literature, recent studies in vocational and higher education provide relevant comparisons. For instance, Pianda et al. (2024) emphasized that internship quality and mentorship strongly predict vocational students’ employability, which echoes our finding that internship experience enhances aviation students’ professional identity and subsequent motivation. Likewise, Feng et al. (2023) demonstrated that internship satisfaction and fulfillment of the psychological contract positively influence graduates’ career identity and engagement, suggesting that psychological mechanisms such as trust and value congruence also underlie the identity-building process in our sample. Similarly, Zhu et al. (2024) revealed that professional identity and intrinsic motivation jointly promote innovative ability among nursing interns, further confirming that identity not only sustains motivation but also stimulates proactive professional behavior. Together, these studies reinforce that identity-based mechanisms are consistent across disciplines and highlight the generalizability of Identity-Based Motivation Theory to the aviation context.

The results of this study provide clear evidence that internship experience influences aviation students’ motivation and professional attitude primarily through the development of professional identity. While internship quality demonstrated a positive total effect on both outcomes, the direct path to motivation remained significant whereas the direct path to professional attitude became non-significant once identity was included as a mediator. This indicates a partial mediation effect for motivation and a full mediation effect for professional attitude. In other words, internships shape students’ motivational beliefs directly to some extent, but their attitudes toward the profession are almost entirely dependent on the degree to which they internalize a professional identity. These findings suggest that it is not internship exposure alone that produces meaningful change, but the extent to which students begin to see themselves as members of the aviation profession.

These results align with findings in nursing (Johnson et al., 2012), teacher education (Glutsch and König, 2019), and hospitality management (Feng et al., 2023), where identity formation has been shown to mediate the impact of practicum experiences on career engagement. However, few studies in aviation education have empirically validated this mechanism. Previous research often assumed that internships improve outcomes directly through skill acquisition or workplace familiarity (Pianda et al., 2024). In contrast, our findings indicate that identity, not skill alone, is the psychological pathway through which experiential learning translates into long-term career commitment. This expands on the work of Lent and Brown (2013), who suggested identity may operate as a motivational resource, by demonstrating that identity fully explains attitudinal outcomes in aviation students. It also provides empirical support for Chao and Yizhou’s (2025) proposition that professional identity is the missing link between internship design and workforce retention in aviation contexts.

This study advances both Social Cognitive Career Theory (SCCT) and Identity-Based Motivation Theory by empirically demonstrating that professional identity is not merely a byproduct of training, but a functional mediator that transforms external experiences into internalized motivation. Unlike traditional models that position internships as direct drivers of outcomes, our results reveal that identity serves as the core mechanism that enhances persistence, perceived meaning, and affective attachment to the profession. This suggests that identity should be treated as a central construct in aviation education research and integrated into models of employability, talent development, and workforce retention.

## 6 Conclusion

This study provides empirical evidence that professional identity is a key psychological mechanism through which internship experiences shape aviation students’ career motivation and attitude. By testing a structured mediation model (see Figure 1), we found that the effect of internship experience on both motivational and attitudinal outcomes is largely indirect, operating primarily through the development of a strong professional identity.

**Figure 2:**
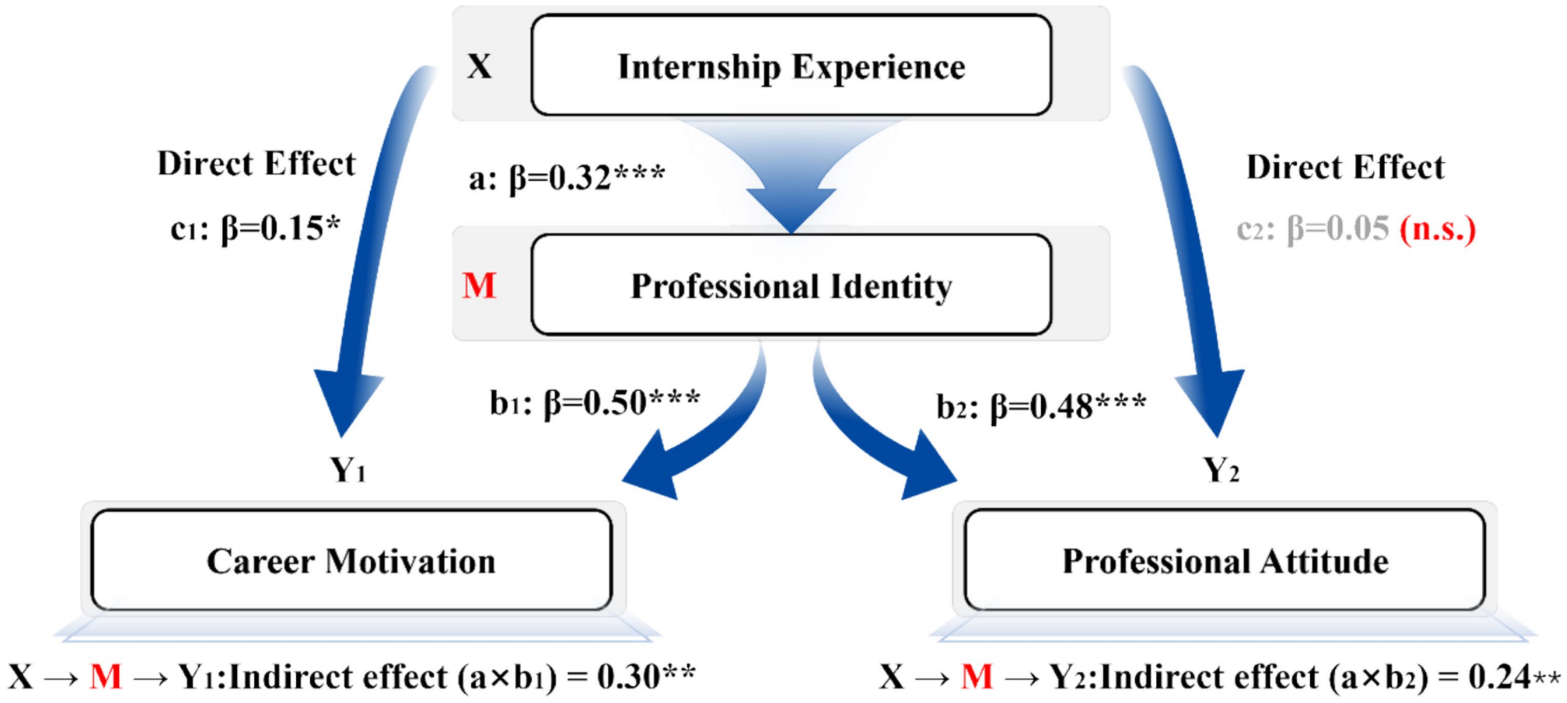
Correlation matrix and structured equation model diagram. Indirect effects (unstandardized, 5,000 bootstrap samples): on Motivation = 0.30 [0.18, 0.45]; on Attitude = 0.24 [0.12, 0.40].

As illustrated in Figure 2, internship experience significantly predicted professional identity (*β* = 0.32*), which in turn strongly predicted both career motivation (*β* = 0.50***) and professional attitude (*β* = 0.48***). Importantly, after accounting for the mediating role of professional identity, the direct effect of internship experience on motivation remained significant (*β* = 0.15*), indicating a partial mediation. However, its direct effect on professional attitude became non-significant (*β* = 0.05, n.s.), demonstrating a full mediation pathway. This pattern reveals that internship experience does not exert its influence on motivational and attitudinal outcomes solely through direct exposure to the work environment, but rather through the psychological process of identity formation.

The indirect effects were confirmed by bootstrap analysis (5,000 samples), showing significant mediation effects on both motivation (indirect effect = 0.30, 95% CI [0.18, 0.45]) and attitude (indirect effect = 0.24**, 95% CI [0.12, 0.40]). These results clearly demonstrate that professional identity is not just a correlate of student outcomes, but a causal psychological mechanism through which experiential learning is transformed into long-term career motivation and positive professional attitudes.

This finding is theoretically significant, as it advances Identity-Based Motivation Theory by providing empirical evidence from the aviation education context. It shows that identity formation is the key driver that translates internship participation into internalized career goals and affective commitment. Practically, this suggests that internship programs should go beyond technical skill training and be intentionally designed to foster identity development through reflective practice, role modeling, and professional socialization. In other words, what students become through internships is more important than what they merely do during internships.

For aviation educators, curriculum developers, and airline HR practitioners, these findings highlight a critical shift in how internship programs should be conceptualized. Rather than viewing internships merely as opportunities for skill acquisition, our results demonstrate that their long-term effectiveness lies in shaping students’ professional identity. The fact that the direct effect of internship experience on professional attitude became non-significant once identity was considered indicates that identity formation is not a peripheral outcome—it is the central psychological mechanism through which experiential learning translates into motivation and commitment.

This means that investing in identity-supportive internships is not simply a training strategy, but a strategic talent development mechanism. Structured reflection, meaningful role modeling, and social recognition during internships are essential in helping students internalize their future professional role. As the aviation industry faces global workforce shortages and retention challenges, supporting the development of a strong professional identity may be just as important—if not more important—than technical training alone.

### 6.1 Limitations and future directions

Several limitations should be taken into consideration when interpreting these findings. One important limitation concerns the cross-sectional nature of the study. Although the mediation effects were statistically significant, the data were collected at a single time point, which means that the temporal ordering of internship experience, identity formation, and outcome variables cannot be fully established. Longitudinal designs would be better suited to validate the developmental process implied in the model. Another issue relates to the sample characteristics. Participants were drawn from three institutions in Northeast China, which offers valuable insights into this specific educational context, yet limits the extent to which the results can be generalized to other regions or to in-service aviation professionals. Replication with more diverse samples would strengthen the external validity of the conclusions.

A further consideration is that all constructs were assessed using self-reported measures. Although this approach is common in psychological and educational research, it may introduce response bias or inflate associations due to shared method variance. Future research could incorporate supervisor ratings, behavioral indicators, or qualitative reflections to enrich the measurement of identity and career outcomes.

Moreover, the present study focused on professional identity as a single mediating mechanism. While this was theoretically justified, internships may also influence students through other psychological processes such as self-efficacy, perceived organizational support, or emotional engagement. Exploring these additional pathways would offer a more comprehensive understanding of how experiential learning contributes to professional development in aviation education.

## Data Availability

The original contributions presented in the study are included in the article/Supplementary material, further inquiries can be directed to the corresponding author/s.
