# Supplementary material for: The impact of internship experience on professional identity, motivation, and attitude among aviation service majors: a cross-sectional empirical study
Source: Front Psychol. 2025 Nov 19;16:1661068. doi: 10.3389/fpsyg.2025.1661068 (PMC12672470; doi:10.3389/fpsyg.2025.1661068)
Supplement: Supplementary file 1 [file Supplementary_file_1.docx]

# Part 1: Demographics & Background

Q1. Year of Study:

- Year 1
- Year 2
- Year 3
- Year 4

Q2. Gender:

- Male
- Female

Q3. Before entering college, how many years have you actively studied aviation service (e.g., self-study, training courses)?

- 1 years
- 2 years
- More than 3 years

Q4. Have you participated in any aviation-related academic activities (e.g., competitions, seminars, research projects)?

- Yes
- No

Q5. Have you received specialized training related to aviation service (e.g., cabin crew training, ground service training, emergency procedures)?

- Yes
- No

Q6. Have you obtained or passed any aviation-related professional certifications or examinations?

- CAAC Cabin Crew Certificate
- ICAO English Level 4+
- Other (please specify)
- None

Q7. Do you have aviation service internship or work experience (e.g., airline internship, airport service, ground staff)?

- Yes
- No

Q8. Do you plan to pursue a career as a flight attendant or aviation service professional in the future?

- Yes
- No

**Q9. What influenced your decision to study Aviation Service Management?**

（Multiple choice）

- Personal interest
- Parental/Teacher encouragement
- Career prospects 职
- Academic background (e.g., aviation-related education, high school aviation clubs)
- Global opportunities (study/work abroad in aviation)
- Social environment (friends/peers/role models in aviation)
- Media exposure (movies, TV, social media, books about aviation service)
- Educational requirements (university admission, scholarship, targeted recruitment)
- Bilingual/Multilingual background
- Technology & digital influence (online platforms, aviation games, aviation forums)
- Other (please specify)

# Part 2: Professional Identity, Attitude in Aviation Service Management

Please indicate how much you agree with each statement about your experience and aspirations in aviation service management.

（1=Strongly Disagree，2=Disagree，3=Neutral，4=Agree，5=Strongly Agree）

1. In-depth study in aviation service management has prepared me to become a qualified aviation service professional.
2. I worry that my understanding of aviation service procedures and relevant knowledge is insufficient for working in the field.
3. I feel confident in my ability to work professionally in aviation service roles.
4. I feel that the demands of working in aviation service might outweigh its rewards.
5. I think working in aviation service will be a fulfilling and meaningful career.
6. I feel uncertain about whether working in aviation service suits my personality and skills.
7. I believe improving my aviation service knowledge and skills is key to becoming an excellent professional.
8. I am concerned that working in aviation service may limit my future career development.
9. I am passionate about working in aviation service to provide excellent experiences for passengers.
10. I sometimes doubt whether I am fully prepared for the challenges of working in aviation service.
11. Working in aviation service may meet my long-term career aspirations.

# Part 3: Career Motivation in Aviation Service Management

Please indicate how much you agree with the following statements about your motivation to pursue a career in aviation service management.

（1=Strongly Disagree，2=Disagree，3=Neutral，4=Agree，5=Strongly Agree）

1. I want to share my passion for the aviation service industry with future passengers and colleagues.
2. I am pursuing a career in aviation service due to the influence of my family and/or teachers.
3. I chose a career in aviation service because it aligns with my personal interests and values.
4. The high demand for aviation service professionals influenced my decision to pursue this career.
5. Learning about aviation service management and related skills has made me more interested in this field.
6. Achieving success or recognition in aviation service studies or internships (e.g., awards, certificates) has encouraged me in this field.
7. Working in aviation service allows me to continue exploring my passion for the industry.
8. The job security and stability of aviation service is one of the key reasons for my career choice.
9. I enjoy the sense of achievement that comes from helping others progress through my work in aviation service.
10. I believe the aviation service industry offers opportunities for professional growth and recognition.

# Part 4: Open-ended Questions on Aviation Service Management

Please answer the following questions in as much detail as possible, helping us better understand your thoughts and experiences.

Q12. Attitudes Toward Aviation Service Careers

In what ways do you think your current knowledge, skills, or experience influence your confidence in pursuing a career in aviation service?

Q13. Influential Aviation Service-related Experiences

Can you describe a specific learning or internship experience that has significantly shaped your attitude toward choosing aviation service as your career?

Q14. Motivation for Choosing Aviation Service Management

What personal interests or values motivate you to pursue a career in aviation service? How do they align with your career goals?

Q15. Key Factors Influencing Your Career Decision

What main reasons influenced your decision to pursue a career in aviation service? Please elaborate on any personal, academic, or external factors that shaped your choice.
